# Gut microbiome profiling of a rural and urban South African cohort reveals biomarkers of a population in lifestyle transition

**DOI:** 10.1186/s12866-020-02017-w

**Published:** 2020-10-31

**Authors:** O. H. Oduaran, F. B. Tamburini, V. Sahibdeen, R. Brewster, F. X. Gómez-Olivé, K. Kahn, S. A. Norris, S. M. Tollman, R. Twine, A. N. Wade, R. G. Wagner, Z. Lombard, A. S. Bhatt, S. Hazelhurst

**Affiliations:** 1 Sydney Brenner Institute for Molecular Bioscience, University of the Witwatersrand, Johannesburg, South Africa; 2 Department of Genetics, Stanford University, Stanford, CA USA; 3 Division of Human Genetics, National Health Laboratory Service, and School of Pathology, Faculty of Health Sciences, University of the Witwatersrand, Johannesburg, South Africa; 4 School of Medicine, Stanford University, Stanford, CA USA; 5 MRC/Wits Rural Public Health and Health Transitions Research Unit (Agincourt), School of Public Health, Faculty of Health Sciences, University of the Witwatersrand, Johannesburg, South Africa; 6 INDEPTH Network, East Legon, Accra, Ghana; 7 SAMRC Developmental Pathways for Health Research Unit, Department of Paediatrics, University of the Witwatersrand, Johannesburg, South Africa; 8 School of Human Development and Health, University of Southampton, Southampton, UK; 9 Department of Medicine (Hematology), Stanford University, Stanford, CA USA; 10 School of Electrical and Information Engineering, University of the Witwatersrand, Johannesburg, South Africa

**Keywords:** 16S, African microbiome, South African microbiome, Obesity, Transitional microbiome, Epidemiological transition

## Abstract

**Background:**

Comparisons of traditional hunter-gatherers and pre-agricultural communities in Africa with urban and suburban Western North American and European cohorts have clearly shown that diet, lifestyle and environment are associated with gut microbiome composition. Yet, little is known about the gut microbiome composition of most communities in the very diverse African continent. South Africa comprises a richly diverse ethnolinguistic population that is experiencing an ongoing epidemiological transition and concurrent spike in the prevalence of obesity, largely attributed to a shift towards more Westernized diets and increasingly inactive lifestyle practices. To characterize the microbiome of African adults living in more mainstream lifestyle settings and investigate associations between the microbiome and obesity, we conducted a pilot study, designed collaboratively with community leaders, in two South African cohorts representative of urban and transitioning rural populations. As the rate of overweight and obesity is particularly high in women, we collected single time-point stool samples from 170 HIV-negative women (51 at Soweto; 119 at Bushbuckridge), performed 16S rRNA gene sequencing on these samples and compared the data to concurrently collected anthropometric data.

**Results:**

We found the overall gut microbiome of our cohorts to be reflective of their ongoing epidemiological transition. Specifically, we find that geographical location was more important for sample clustering than lean/obese status and observed a relatively higher abundance of the *Melainabacteria*, *Vampirovibrio*, a predatory bacterium, in Bushbuckridge. Also, *Prevotella*, despite its generally high prevalence in the cohorts, showed an association with obesity. In comparisons with benchmarked datasets representative of non-Western populations, relatively higher abundance values were observed in our dataset for *Barnesiella* (log_2_fold change (FC) = 4.5)*, Alistipes* (log_2_FC = 3.9), *Bacteroides* (log_2_FC = 4.2), *Parabacteroides* (log_2_FC = 3.1) and *Treponema* (log_2_FC = 1.6), with the exception of *Prevotella* (log_2_FC = − 4.7).

**Conclusions:**

Altogether, this work identifies putative microbial features associated with host health in a historically understudied community undergoing an epidemiological transition. Furthermore, we note the crucial role of community engagement to the success of a study in an African setting, the importance of more population-specific studies to inform targeted interventions as well as present a basic foundation for future research.

**Supplementary information:**

**Supplementary information** accompanies this paper at 10.1186/s12866-020-02017-w.

## Background

There have been relatively few studies of the human gut microbiome in Africa, with most reported studies to date focusing on the extremes of non-Western traditional hunter-gatherer and agriculturalists African populations, as well as children with nutritional deficiencies [1–7]. A consistent finding of these studies is the inverse relationship in the relative abundance of *Bacteroides* and *Prevotella* genera of the *Bacteroidetes* phylum. *Prevotella* is associated with plant-based diets predominantly in non-Western populations, whereas increased relative abundance of *Bacteroides* is thought to result from animal fat- and protein-based diets [3, 7–11]. These studies have been vital in providing great insight into the microbiome of traditional African populations and pioneering the efforts of microbiome studies on the continent. It is important to note that across most of sub-Saharan Africa, although the lifestyle has been dominantly agricultural for at least 1000 years [12], relatively few people practice hunter-gatherer or pastoralist lifestyles. However, over the last 50 years in particular, there has been an epidemiological transition toward more industrialized and sedentary lifestyles, that has had significant impact on many Africans.

The role of the microbiome in areas of public health has also been a study focus area on the African continent. These include nutrition, vaccine response efficacy, the impact of antibiotics, mental health and human immunodeficiency virus (HIV) [13–16]. Obesity, a growing health burden [17] on the African continent, has received comparably less attention from microbiome researchers. In a ground-breaking effort, however, the first study on type 2 diabetes (T2D), a comorbidity of obesity, on a sub-Saharan African population [18], provided some insight into the association of gut microbial profiles to T2D in individuals in an urban African setting. The dramatic increase in the prevalence of obesity has been attributed, in part, to the ongoing shift on the continent towards more Westernized practices, such as the consumption of more animal-based and processed products with increasing physical inactivity [19–21], further complicating the existing challenge of malnutrition facing the continent [22, 23]. This is reflected in an analysis of demographic and health survey data from 24 African countries [17] where the prevalence of overweight and obesity among women increased in all 24 countries with either a doubling or tripling in the incidence of obesity reported in 50% of the surveyed countries. Pertinent to this study are the statistics indicating black South African women to have the highest prevalence of obesity (42%) within sub-Saharan Africa [24] with general continental body mass index (BMI) trends showing a decline in the underweight population with a concomitant increase in the overweight and obese population [25–27]. The implication of this is the potential increase in the prevalence of comorbidities including diabetes and other cardiometabolic diseases augmenting the health and economic burden in African societies [28–30]. Reports have also alluded to the influence of the growing globalization trend, its concurrent urbanization and consequent dietary implications on otherwise rural areas in South Africa [31–36]. This is reflected in the increasing numbers and proximity of supermarkets and fast food outlets in these areas [31, 33].

Globally, several studies have focused on understanding the apparent dysbiosis observed in obesity [37, 38]. African populations have, however, been understudied in these efforts. Consequently, there is a paucity of data within Africa comparing the gut microbiota of obese individuals to their leaner counterparts. This is crucial, as differences in dietary and environmental exposures may render findings in non-African populations poorly generalizable to the African context, especially with the ongoing epidemiological transition in Africa [4, 39, 40].

Here, we present a study that investigated the gut microbial composition of two South African cohorts with some insight into the microbial compositional differences between obese and lean individuals in the changing microbiota landscape. South Africa, with its diverse ethnolinguistic groups, presents a unique opportunity to study the effects of this continent-wide transition on the gut microbiome. With obesity being an established risk factor in cardiometabolic diseases, understanding the differences observed between obese and lean individuals in this setting could prove critical to improving our understanding of its association to the pathogenesis of the disease.

This pilot study was nested in the AWI-Gen project [41], a part of the Human Heredity and Health in Africa (H3Africa) [42] initiative. AWI-Gen is a collaborative effort, with participants in six sites across four African countries, established to assess genomic and environmental factors that influence cardiometabolic diseases risk, with the aim of informing treatment and intervention strategies. The study focused on characterizing the gut microbiome of female adults, with body mass indices spanning the lean and obese range, from two cohorts comprising communities across two South African provinces, Gauteng and Mpumalanga, representative of relatively urban and transitioning rural lifestyles respectively. These cohorts are managed by established health and demographic surveillance sites (HDSS) in partnerships with the University of the Witwatersrand (Wits) and the Medical Research Council (MRC) of South Africa. The Agincourt HDSS [35] in Mpumalanga encompasses a collection of rural communities in the Bushbuckridge municipality undergoing rapid epidemiological changes which may allow for some of the areas to be classified as peri-urban. The Developmental Pathways for Health Research Unit (DPHRU) in Gauteng, on the other hand, is focused on Soweto, a highly urbanized area in the Johannesburg metropolitan area. Soweto has been urbanized for many generations even though in-migration remains at a high level.

In this study, we performed 16S rRNA gene analysis of the gut microbiome of 170 female individuals in Bushbuckridge and Soweto. We evaluated the overall microbial composition of the sampled data to improve our knowledge of the general microbiota landscape of these representative cohorts and assessed compositional differences in the microbiome between lean and obese individuals, using BMI values, within and between Bushbuckridge and Soweto. We also provide insight into the feasibility of such studies in rural communities whilst highlighting the importance of community engagement to this effort.

## Results

### Participant recruitment and study cohort

With ethics approval from the Human Research Ethics Committee (Medical) of the University of the Witwatersrand (M160121) and the Provincial Health Research Committee of the Province of Mpumalanga (MP2017TP22851), 132 female individuals from Bushbuckridge (24.8398° S, 31.0464° E) and 58 from Soweto (26.2485° S, 27.8540° E) were recruited for the study. However, only 170 participant samples (Bushbuckridge: 119, Soweto: 51) were included in the study due to confounding factors to the focus of this pilot (18 HIV-positive samples and two samples with collection irregularities were excluded). The age and BMI distribution of the cohorts are shown in Table 1.

**Table 1:** Age and BMI distribution of cohorts

	Mean ± SD	Median	Range
**Age** (years)			
Bushbuckridge	55.50 ± 7.77	55	43 - 72
Soweto	54.10 ± 5.86	54	43 - 64
**BMI** (Kg/m^2^)			
Bushbuckridge	32.6 ± 7.95	31.17	21.23 - 58.97
Soweto	36.05 ± 9.23	36.52	20.43 - 58.62

### Pre-processing and quality control

**Fig. 1:**
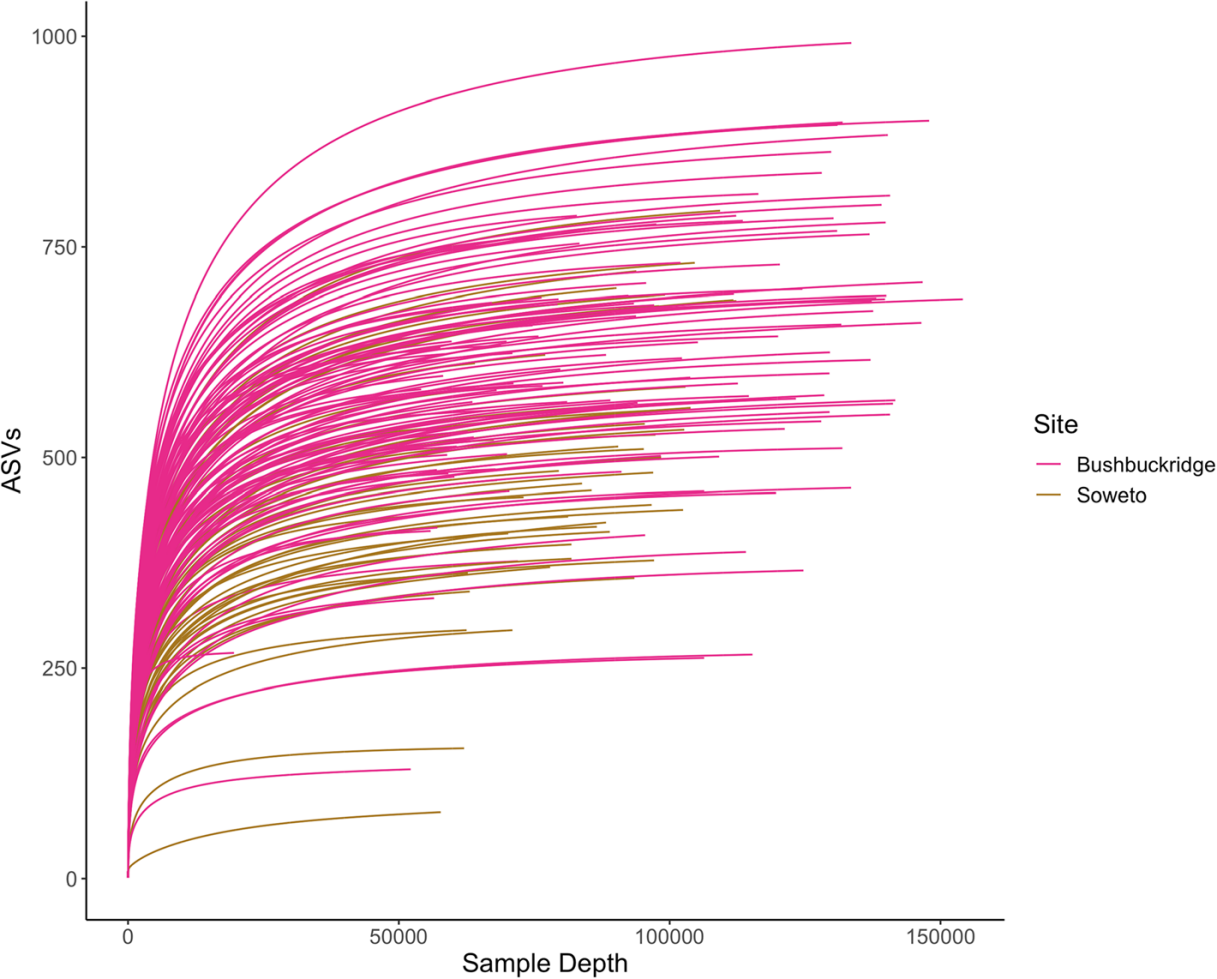
Rarefaction curve of sampled data. This figure shows all 170 of the sampled across the Bushbuckridge and Soweto cohorts

This was primarily done with the DADA2 pipeline [43]. 16S rRNA gene sequencing was performed with primers to the V3 and V4 regions. A total of 15,839,081 sequences were obtained from the 170 samples after quality control. The sequence depths ranged from 2 to 154,124 reads per sample (Supplementary Table 1), with a mean of 93,171.06 ± 2275.40 and a median of 93,066, resulting in a total of 10,088 unique amplicon sequence variants (ASVs) with redundant taxonomies. As a result of relatively low sampling depths, the spread of the read depths and the likelihood that the richness of the samples was not fully observed at their sequenced depths, three samples with fewer than 19,560 reads were excluded from downstream analyses (Fig. 1). The implication of this exclusion is an overall minimum sequence depth of 50,812 reads for the 167 samples. The dataset was further pruned to remove taxa not seen more than three times in at least 5 % of the 167 samples in order to protect against ASVs with small mean and trivially large coefficients of variation [44]. This resulted in 1688 ASVs being used as input for beta diversity and the differential abundance analysis implemented with DESeq2 [45]. The taxonomies associated with the corresponding ASVs accounted for two kingdoms (*Archaea* and *Bacteria*) resulting in 14 phyla, 25 classes, 30 orders, 54 families, 124 genera and 111 species, with unclassified ASVs also detected at all but the kingdom levels (Table 2). These numbers represent non-redundant taxa.

**Table 2:** Distribution of taxonomic classification of filtered ASVs in sampled South African pilot dataset

Taxa Level	Classified ASVs	Associated Taxa	% Unclassified ASVs
Kingdom	1,688	2	0.00%
Phylum	1,668	14	1.18%
Class	1,638	25	2.96%
Order	1,628	30	3.55%
Family	1,444	54	14.45%
Genus	1,114	124	34.00%
Species	161	111	90.46%

### Microbial community richness estimates and differences

With the majority of diversity metrics being sensitive to varying sequencing depths across samples [46], rarefaction was done at a read depth of 50,800 to maximize the capture of the observed microbial taxa richness in the cohort. This cut-off was chosen based on the spread of the read depths as visualized in the rarefaction plot in Fig. 1. The rarefied dataset was used for the alpha diversity analyses.

#### Site differences

**Fig. 2:**
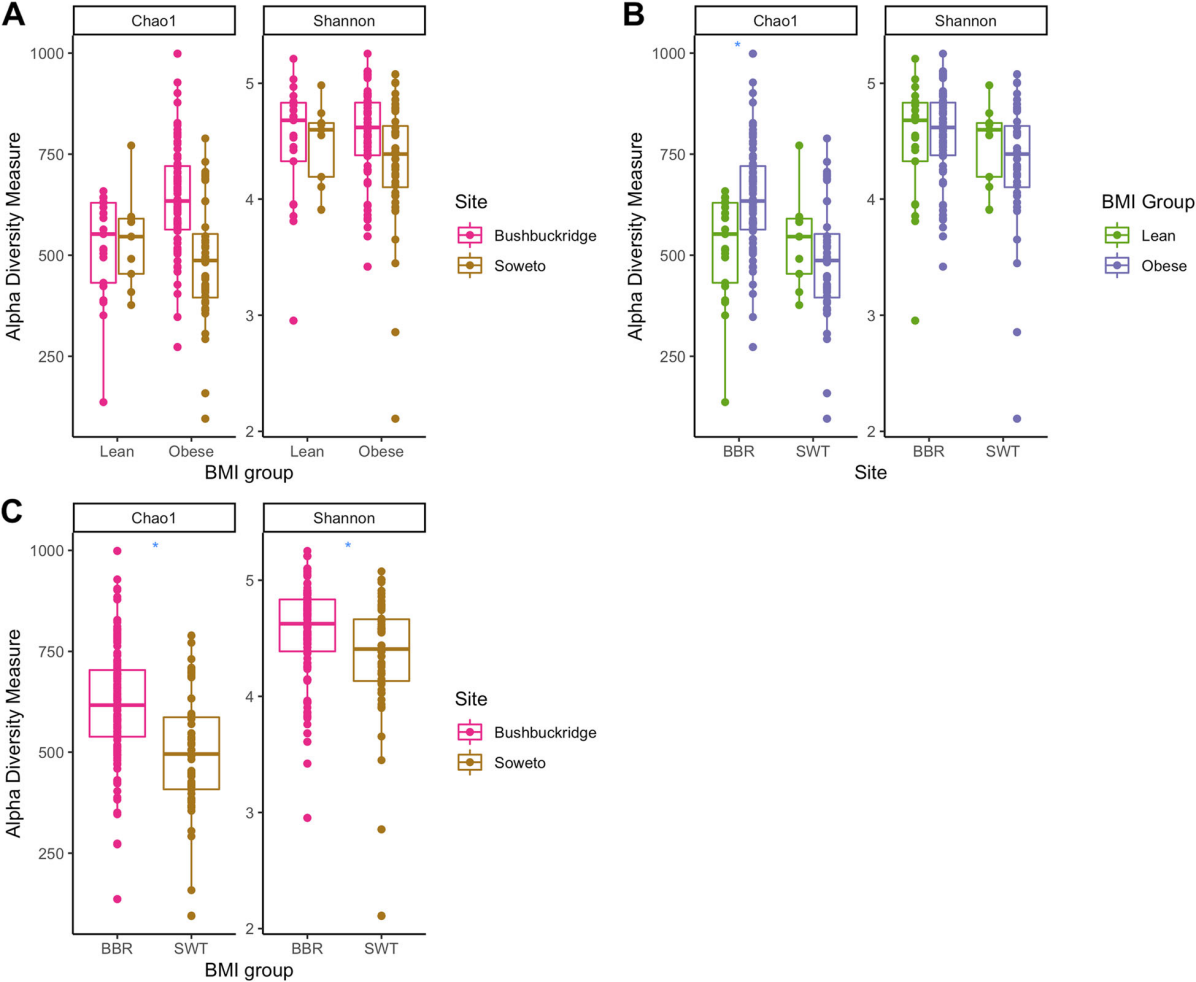
Boxplots of Shannon and Chao1 alpha diversity measure estimates. Alpha diversity comparisons of lean and obese samples: (**a**) cohort-wide and (**b**) site-specific. Overall study cohort differences are shown in (**c**). ‘*’ indicates a statistically significant difference as measured by the Wilcoxon rank sum test with a *p*-value of < 0.05

**Fig. 3:**
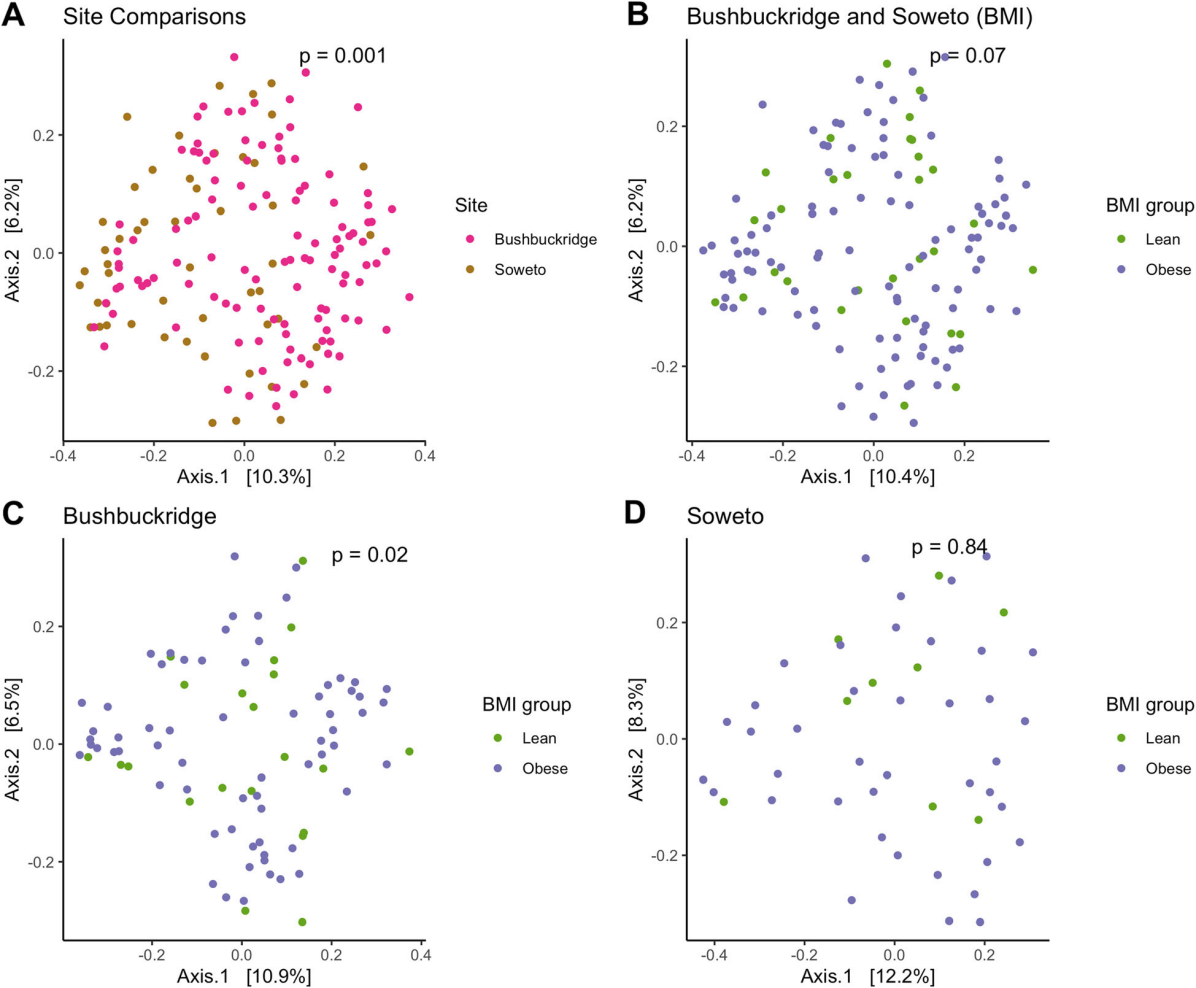
Beta diversity PCoA plots with Bray-Curtis dissimilarity measure. Combined Bushbuckridge and Soweto datasets indicating differences in (**a**) Cohort-wide and (**b**) Lean vs obese categories. Site-specific lean and obese sampled data in (**c**) Bushbuckridge and (**d**) Soweto. Inset *p*-values resulted from PERMANOVA analysis between compared groups

In a cohort-wide comparison to evaluate overall differences between the Bushbuckridge and Soweto sites irrespective of BMI status, statistically significant *p*-values were observed for alpha diversity measures of both Shannon [47] (*p* = 0.012) and Chao1 richness (*p* < 0.001) [48] (Fig. 2), and the Bray-Curtis dissimilarity measure (*p* = 0.001), visualized in principal coordinate analysis (PCoA) [49] plots (Fig. 3). We find that geographical location was more important for sample clustering than lean/obese status. The PCoA plots also present a moving divide between rural Bushbuckridge and urban Soweto. This appears to reflect a transitional state possibly owing to gradual lifestyle and dietary changes.

#### BMI differences

In evaluating the potential diversity across BMI categories, Shannon diversity, a measure of richness and evenness, for the lean and obese groups in Bushbuckridge (Fig. 2b) were 4.49 ± 0.53 and 4.56 ± 0.39, respectively. The exclusion of an apparent outlier in the Bushbuckridge lean group resulted in a Shannon index of 4.56 ± 0.41 in that group. The corresponding estimates for Soweto were 4.49 ± 0.34 (lean) and 4.30 ± 0.56 (obese). The differences between the lean and obese groups did not reach statistical significance as indicated by the non-parametric Wilcoxon rank sum test evaluating the Shannon diversity values between both groups (*p* = 0.85 and 0.45 for Bushbuckridge and Soweto respectively). Beta diversity measurements (Fig. 3), however, showed statistically significant differences between the lean and obese groups in Bushbuckridge with calculated Bray-Curtis distances using the permutational analysis of variance (PERMANOVA) test (*p* = 0.02 for Bushbuckridge and *p* = 0.84 for Soweto (Table 3).

**Table 3:** Alpha and beta diversity significance of compared groups. Alpha diversity *p*-values were calculated with pairwise Wilcoxon rank sum test. Bray-Curtis diversity *p*-values were calculated with PERMANOVA

Sample Distribution	Group Comparisons	Shannon ***p***-values	Chao1 ***p***-values	Bray-Curtis ***p***-values
Bushbuckridge and Soweto	Lean vs Obese	0.72	0.06	0.07
Bushbuckridge and Soweto	Lean vs Lean	0.69	0.72	0.01
Bushbuckridge and Soweto	Obese vs Obese	0.01	< 0.001	0.001
All samples	Bushbuckridge vs Soweto	0.01	< 0.001	0.001
Bushbuckridge	Lean vs Obese	0.85	0.001	0.02
Soweto	Lean vs Obese	0.45	0.33	0.84

### Taxonomic analyses

**Fig. 4:**
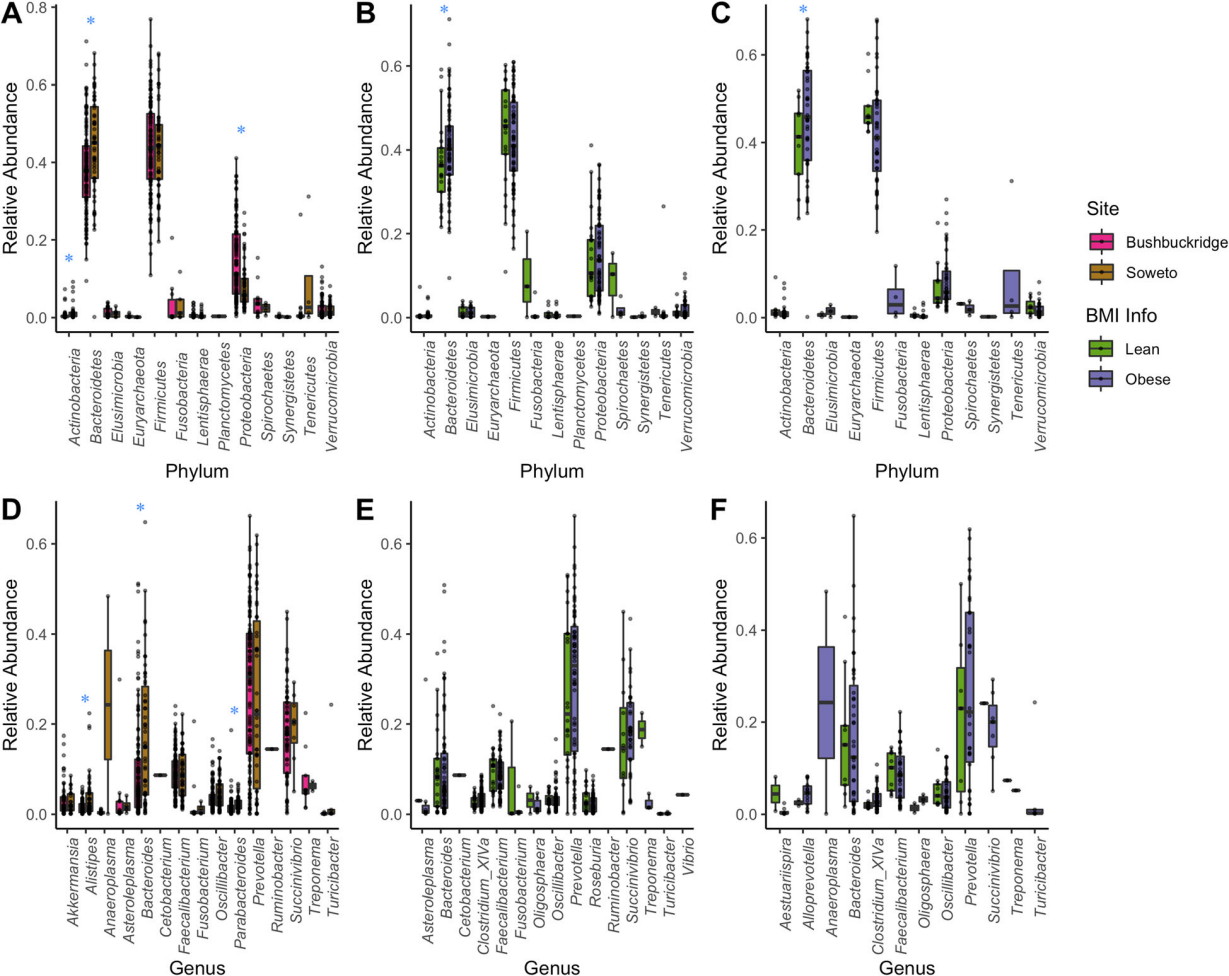
Taxonomic profiles of the gut microbiome of the sampled South African dataset. Phylum level relative abundance values are depicted in the boxplots in (**a**) Combined Bushbuckridge and Soweto cohorts, (**b**) Bushbuckridge and (**c**) Soweto. The corresponding genera abundance levels are depicted in **d, e and f** respectively. ‘*’ indicates a statistically significant difference as measured by the Kruskal-Wallis rank sum test with a *p*-value of < 0.05

Overall, *Firmicutes* (43.7% ± 11.8%), *Bacteroidetes* (40% ± 12.1%) and *Proteobacteria* (12.5% ± 9.1%) were the dominant phyla observed in the combined gut microbiome data from these two South African cohorts (Fig. 4). Three phyla – *Actinobacteria* (*p* < 0.001), *Bacteroidetes* (*p* = 0.001), *Proteobacteria* (*p* < 0.001) and three genera – *Alistipes* (*p* < 0.001). *Bacteroides* (*p* < 0.001) and *Parabacteroides* (*p* < 0.001), showed significant differences in relative abundance values between the two cohorts based on Kruskal-Wallis (KW) *p*-values.

The noticeably higher relative *Bacteroides*’ abundance (17.1% ± 15.%) observed in urban Soweto in comparison with Bushbuckridge (9.8% ± 11.4%) together with the presence of *Alistipes*, *Anaeroplasma* and *Barnesiella* amongst the most abundant genera is in line with the association of these taxa with non-Western populations in literature (Fig. 4d, e and f) [10, 40, 50]. These associations have been hypothesized to be driven by diet [3, 51, 52]. Of note, within-cohort taxonomic comparisons between lean and obese individuals did not reveal any significant differences at both phyla and genera levels.

### Microbial compositional analyses

To better understand the contribution of lifestyle to microbiome composition in this pilot study, the DESeq2 [45] method was applied to further evaluate potential compositional differences in the South African cohorts. To accomplish this at site level, the data was first sub-setted to exclude the intermediate, overweight samples, while keeping only the lean (Bushbuckridge: 21, Soweto: 9) and obese samples (Bushbuckridge: 66, Soweto: 40).

#### Cohort-wide analysis

**Fig. 5:**
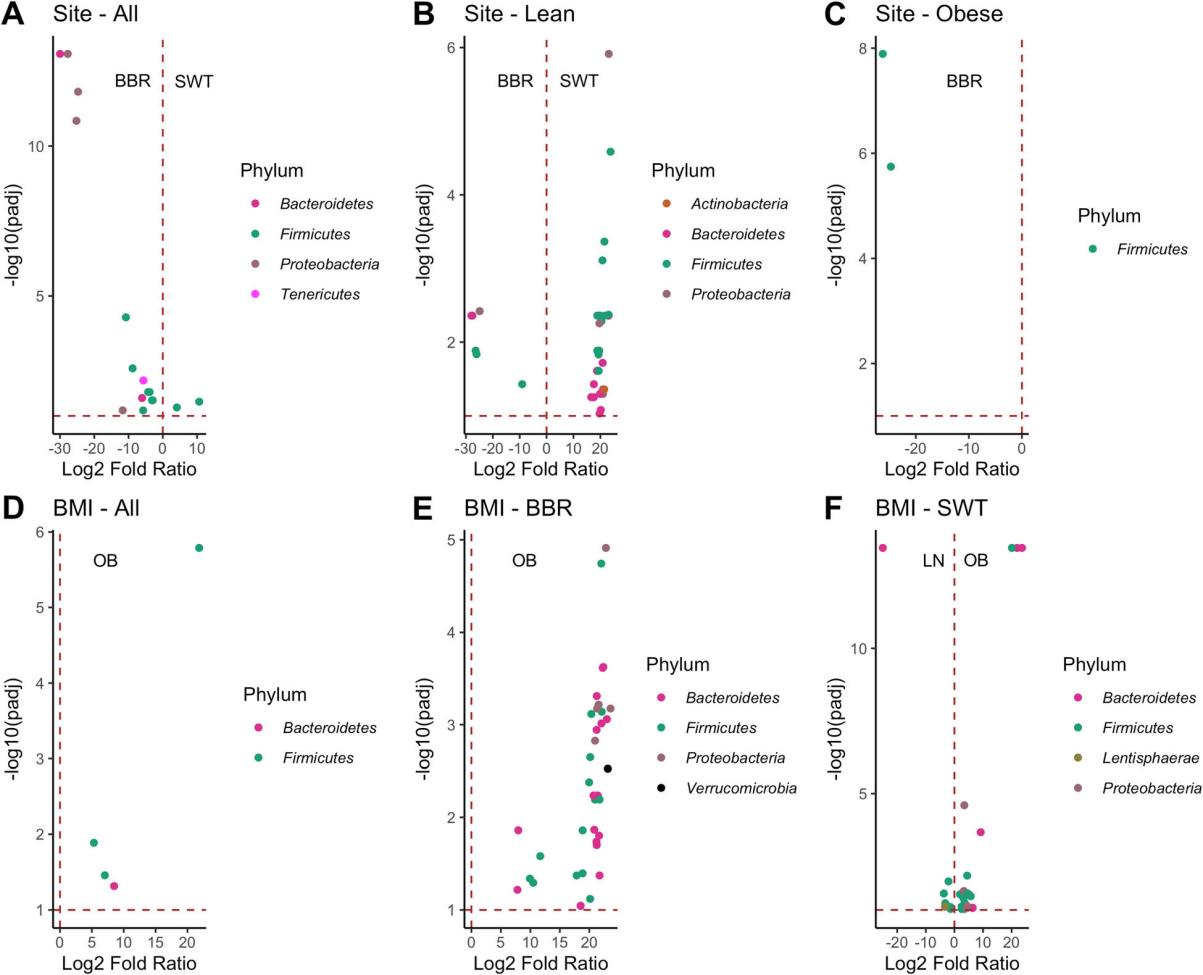
Differential abundance comparison volcano plots of ASVs significantly abundant in Soweto (SWT) vs Bushbuckridge (BBR) in (**a**) Combined dataset, (**b**) Lean samples and (**c**) Obese samples. ASVs significantly abundant in obese (OB) vs lean (LN) samples are shown in (**d**) Combined dataset, (**e**) Bushbuckridge and (**f**) Soweto. The horizontal dashed line indicates a threshold of Benjamini-Hochberg-adjusted *p* < 0.1

Differential abundance analysis revealed a general high prevalence of *Prevotella* in the South African dataset. Also present in the cohorts were *Phascolarctobacterium* and *Vampirovibrio*, which was observed primarily in the Bushbuckridge cohort (Fig. 5a and e; Supplementary Tables 2A and E). *Alistipes*, a genus associated with Western populations, showed significantly higher differential abundance in Bushbuckridge (Fig. 5a; Supplementary Table 2A). Some of the other taxa associated with Bushbuckridge include the flavonoid-degrading *Flavonifractor, Parasutterella, Gemmiger,* and *Dialister* [48] (Fig. 5a and c; Supplementary Tables 2A and C). Soweto samples, on the other hand, showed a significant enrichment in *Bifidobacterium*, the oxalate-metabolizing *Oxalobacter* [53, 54], *Barnesiella, Acetanaerobactrium*, *Roseburia, Escherichia/Shigella* and *Streptococcus* (Fig. 5a, b and f; Supplementary Tables 2A, B and F).

Comparing the microbiomes of the combined obese groups (Bushbuckridge and Soweto) with their leaner counterparts revealed butyrate-producing *Intestinimonas* [55] *and Prevotella* to be more abundant in the obese category with log_2_fold changes of 5.32 and 8.50 respectively (Fig. 5d; Supplementary Table 2D).

#### Site-specific analysis

Notably, *Prevotella* was found to be associated with obesity. This was clearly observed in Bushbuckridge, where *Prevotella* showed a higher relative abundance in the obese group (Fig. 5d, e and f; Supplementary Tables 2D, E and F). Also observed to be in higher abundance in the Bushbuckridge obese group were 36 ASVs representative of 11 unique genera which include *Prevotella* (12), unclassified genera (10), *Sutterella* (3), *Phascolarctobacterium* (2), *Ruminococcus* (1), *Clostridium_IV* (1), *Alistipes* (1), *Acetanaerobacterium* (1), *Parabacteroides* (1), *Catenibacterium* (1) and *Akkermansia* (1) (Fig. 5e; Supplementary Table 2E). The numbers in parenthesis are the associated ASVs. In Soweto, 24 ASVs, representative of 12 genera, were associated with the obese group while seven ASVs representative of four genera presented higher abundance levels relative to their leaner counterparts. The obese group-associated genera are *Prevotella* (6), *Clostridium_XIVa* (3), *Haemophilus* (3), *Oscillibacter* (2), unclassified genera (2), *Clostridium_XIVb* (1), *Streptococcus* (1), *Escherichia/Shigella* (1), *Ruminococcus* (1), *Sporobacter* (1), *Oxalobacter* (1), *Intestinimonas* (1) and *Parabacteroides* (1). The genera associated with the lean group in Soweto are *Parabacteroides* (1), *Victivallis* (1), *Fusicatenibacter* (1) and unclassified genera (3) (Fig. 5f).

The apparent site-specific association of *Prevotella* to the obese group in Bushbuckridge is in line with literature linking the taxon to obesity [38, 56, 57], although there have also been some contradictory reports [1, 2].

### Marker taxa analyses

A recent meta-analysis examined differences between the gut microbial composition of traditional, rural populations and their more industrialized counterparts from several studies with datasets encompassing 13 developed or industrialized societies and two traditional hunter-gatherer, pre-agricultural communities [3, 4, 8, 58, 59]. The study proposed a marker taxa list distinguishing Western and non-Western bacterial communities. This was corroborated by de la Cuesta-Zuluaga, et al. [60] by the analysis of 16 benchmark datasets with the Bioconductor package, curatedMetagenomicData (cMD) [61]. The cMD is a collection of processed data from whole-metagenome sequencing for thousands of human microbiome samples across different body sites.

**Fig. 6:**
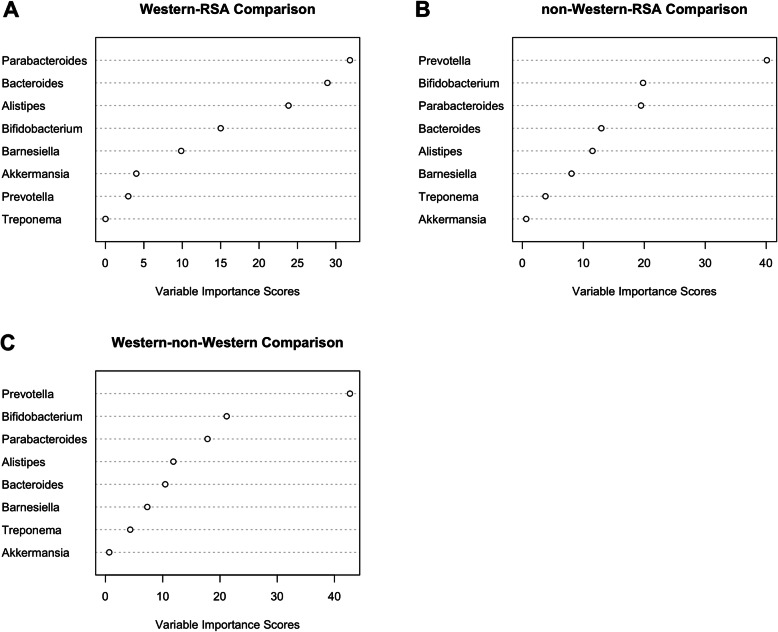
Variance Importance Plot resulting from the Random Forest analysis of proposed Western and non-Western marker taxa abundances in the subsampled curatedMetagenomicData (cMD). Comparisons between the study data (RSA) with (**a**) Western cMD, and (**b**) non-Western cMD. **c** Western versus non-Western cMD comparison

To further evaluate the landscape of our study data with respect to the established population-dependent compositional expectations, we randomly selected 334 individuals from the cMD, 167 of whom were from populations of Western origin and the remaining 167 from traditional non-Western populations to match the number of samples in our dataset. The sampling was done from a total of 23 studies with 1763 samples (1433 Western and 330 non-Western) in the cMD. We compared the abundance values of Western-associated (*Alisitipes*, *Akkermansia*, *Barnesiella*, *Bifidobacterium*, *Bacteroides* and *Parabacteroides*) and non-Western-associated (*Treponema* and *Prevotella*) marker taxa to their corresponding abundance profiles in our dataset. This was done by testing the null hypothesis that the mean ranks of the abundances of these marker taxa were the same in the subsampled cMD and our sampled cohorts using the non-parametric Kruskal-Wallis test. Our results rejected the null hypotheses for all (*p* < 0.001) but three taxa, *Akkermansia*, *Barnesiella* and *Treponema* with *p* > 0.1 when compared to corresponding Westernized datasets. Comparisons with the non-Western dataset, on the other hand, resulted in the rejection of the null hypothesis for all but one taxon, *Treponema* (*p* = 0.52). We found the abundances of *Alistipes*, *Bacteroides*, *Prevotella*, and *Parabacteroides* in our data to be intermediate between the benchmarked Western and non-Western datasets, and the abundance of *Barnesiella* comparable to that in the Western microbiota (Table 5). In addition, Random Forest analysis comparing the South African cohorts to the subsampled cMD presented *Prevotella* and *Parabacteroides* as the most important discriminatory taxa in the non-Western and Western datasets comparisons respectively (Fig. 6a and b). Interestingly, the importance scores associated with each taxon in the classification of the subsampled non-Western cMD with our dataset is comparable to the associated taxa scores in the classification of the cMD’s Western and non-Western datasets (Fig. 6c). Altogether, these results reinforce the notion of a gradually changing microbial composition of the sampled cohort relative to the subsampled curated datasets.

## Discussion

This study aimed to characterize the gut microbiome of two South African cohorts from two sites, about 483 km (300 miles) apart that represent relatively urban and transitioning rural lifestyle and diet-practicing populations, whilst exploring the microbial compositional differences observed in obese and lean individuals. To accomplish this, we collaboratively designed a study with active input from the community, in conjunction with a community advisory group (CAG) at Bushbuckridge. Although the community was familiar with the general research process, the concept of stool donation was relatively unfamiliar [62, 63]. Stool collection for microbiome research purposes had never before been carried out in this population. With prevailing traditional beliefs concerning stool carrying the soul, it was crucial to be sensitive and respectful whilst clearly presenting the importance and proposed usage of the stool samples as well as the aims of the research in understandable language. The recruitment process and sample collection for this study thus relied on extensive community engagement.

DNA extracted from the collected stool samples underwent 16S rRNA gene sequencing. We observed relative abundance levels of Western gut-associated marker taxon, *Barnesiella,* that were comparable to Western populations with intermediate abundance levels for *Alistipes*, *Bacteroides*, *Parabacteroides* and *Prevotella* when compared to the benchmarked datasets (Table 5). Within our cohorts, we found *Vampirovibrio*, a predatory *Melainabacteria* to be present with higher relative abundances in the rural samples and *Prevotella*, despite its generally high prevalence relative to all taxa present in the cohort, to be associated with obesity. Overall, we identified putative microbial features associated with host health and highlight the importance of population-specific considerations in microbiome research. Importantly, we also shed some light on the vital role of engaging the community of interest to the success of such studies in an African setting.

Within our cohorts, microbial composition reflected a transitional state comprising both Western- and non-Western-associated taxa. *Prevotella* and *Treponema* represented the traditional hunter-gatherer taxa. *Phascolarctobacterium*, a propionate and acetate producer that has been shown to exert beneficial effects on its host [64–66], appears to be abundant across both sites. A recent study comparing various industrialized, urban populations to traditional rural societies identified *Phascolarctobacterium* to be the most significant contributing taxa to the non-Western population cluster [64]. A robust meta-analysis study that compared the gut microbiomes of urbanized and pre-agricultural populations also noted it to have relatively low abundance, and in some cases absence, in Western populations [67].

With global research findings on the apparent dysbiosis of the gut microbiome in obesity being inconclusive [38, 39, 68, 69], we sought to evaluate the differences between obese and lean individuals within and between the two study populations. The within site differences were moderate and did not reach statistical significance in Soweto. However, for Bushbuckridge, significant differences were observed for both alpha and beta diversity estimates between the lean and obese groups using Chao1 (*p* = 0.001) and Bray-Curtis (*p* = 0.02) measures. Log_2_ fold changes ranging from 7.81 to 23.60 were observed in the differential abundance analyses of component microbial taxa of the obese samples relative to their leaner counterparts resulting in 11 classified genera. *Sutterella* and *Catenibacterium* which have been previously associated with obesity [70, 71], as well as *Clostridium_IV* were among the differentially abundant taxa in the obese samples in Bushbuckridge. *Oscillibacter* was associated with cohort-wide obesity irrespective of site. This association to obesity has been previously reported in a European cohort [72].

Overall, the lean comparisons showed slightly greater diversity than the obese groups with taxa representative of four different phyla and 14 genera (Fig. 5b and c). The PCoA plots comparing lean and obese individuals (Fig. 3b, c and d) appears to show a divide between samples that may not be entirely driven by BMI categories. It is, however, possible that associations with small effect sizes exist in our sampled cohort that could be detected with larger sampling. Also, as limited demographic and dietary data were collected for this pilot, further exploration is warranted.

Of great interest in the Bushbuckridge cohort was the predatory *Vampirovibrio*. Although not very well-studied in humans to date, *Vampirovibrio* is capable of invading and attacking other bacteria without harming human cells. It has been proposed for further studies in bioremediation [73] to reduce the use of antibiotics. *Melainabacteria*, the phylum to which *Vampirovibrio* belongs [74–76], is generally found to be present in aquatic habitats as well as associated with the guts of herbivorous mammals and humans with predominantly plant-based diets. They are also known to synthesize vitamins B and K, which in addition to their fiber-digesting abilities posits them as beneficial bacteria to their hosts.

Several studies have identified obesity-associated taxa primarily in non-African populations [25, 77, 78] despite these reported connections being inconsistent [1, 2, 72]. The differential abundance, prevalence or presence of microbial taxa across populations may require population-specific associations for relevance, as universal classifications may not necessarily be generalizable. The seemingly ubiquitous presence of *Prevotella* in the sampled cohorts and its association with obesity in Bushbuckridge brings to the fore the role of some *Prevotella* strains as potential pathobionts involved in various human diseases by the promotion of chronic inflammation [79, 80]. Increased abundance of *Prevotella* species at mucosal sites have been linked to several diseases including metabolic disorders and low-grade systemic inflammation [38, 56, 81], a feature associated with obesity. *Prevotella* may thus present as a critical taxon in the obesity pandemic on the African continent. Further in-depth studies to ascertain the influence of its prevalence in a community undergoing such epidemiological transition will be insightful as the beneficial or detrimental effects of *Prevotella* may very likely be dependent on strain variations or its interaction with the prevailing lifestyle and environment [82].

## Conclusions

This study provides us with a foundation to inform future microbiome studies in Africa. A clear outcome of this study was the statistically significant differences in microbial composition observed between the Bushbuckridge and Soweto cohorts with the Bushbuckridge cohort harboring relatively more diverse microbiota. This highlights the difference in stages of the cohorts along the continuum of transition, with the gradual lifestyle and dietary shifts towards more Western practices. Such clarity was not consistently achieved statistically for comparisons between the BMI categories considered. However, moderate differences were observed. This could possibly be attributed to the uneven and sparse sampling of the data especially with the lean category in Soweto. Notwithstanding, the core outcome of this analysis does not seem to have been affected as observed in comparisons between the lean populations of both cohorts. Similarly, a lack of inflated significance in differential abundances between the groups compared support the integrity of the study outcome.

We acknowledge that this study was limited by the unavailability of detailed dietary data at the time of sample collection that may have explained some of the observations and extended the scope of the study. No assumptions were made in this regard with the data presented as is. However, there are published reports on the dietary changes accompanying the urbanization process across rural areas in South Africa [31–33, 36]. Another potential limitation of this study is the aforementioned uneven and sparse sampling of the data, which appears to have been inconsequential on the study outcome. It is important to note that this was a pilot exploratory study that has provided useful insights into the planning and execution of future studies in similar settings.

In broad summary, the compositional taxa of the gut microbiome of the collective ethnolinguistic groups in the cohorts are reflective of an epidemiologically transitional state, and the beneficial or detrimental effects of *Prevotella* are very likely diet- and lifestyle-dependent. Lastly, the largely intermediate abundances of the proposed Western and non-Western distinguishing marker taxa in our data set in comparison with benchmarked datasets substantiates the transitional state of our African cohorts with potential implications for disease pathogenesis and general health status. This accentuates the need for more population-specific studies as findings and translational applications in non-African populations may be poorly generalizable to the African context. Further studies with a larger sampled cohort will be very informative in this regard.

## Methods

### Community engagement

The research team engaged the community in two interactive sessions during this study - the planning phase and post-preliminary analyses on the data resulting from the collected stool samples. A survey was also conducted on the first 100 participants in Bushbuckridge to get their feedback on the process. Prior to the collection of stool samples for the study, there was interaction with the community in conjunction with a CAG at the Agincourt HDSS (Bushbuckridge), the rural site, which gave input into the process to ensure that sample collection methods were sensitive to the community beliefs and applicable to the existing toilet facilities in the area. This group comprised eight community representatives and indunas (village councillors). The meeting discussions were focused on creating awareness on what the project entailed and the importance of such research in the community, as well as on potential concerns and reactions of community members to stool sample collection and the practicality of such endeavor. Also deliberated on was the role of the trained fieldworker in the recruitment process and the available resources (graphical flyers) to clearly communicate the study aims and usage of the collected stool samples in understandable language to potential participants.

The interactive workshop that followed the preliminary data analysis aimed to reiterate the importance of the study, broadly present some of the initial results and very importantly, solicit feedback from the community members and participants. As this was a pilot study, it was important to the research team to gauge the level of understanding of the study post-completion in order to inform future studies in this regard.

### Recruitment and study cohort

This study is nested in the AWI-Gen project, which is a part of the Human, Heredity and Health in Africa (H3Africa) consortium. AWI-Gen explores genetic and environmental factors in cardiometabolic disorders in African populations with six sites across four countries. The recruitment of participants for this study was done at two of the South African sites – the Bushbuckridge area within the Agincourt HDSS, Mpumalanga (rural) and Soweto, Johannesburg, Gauteng (urban).

Participants were randomly selected from the AWI-Gen cohort within the BMI strata defined below and are in the age range of 43–72 years (Table 1). To minimize confounding effects, male and HIV+ participants were excluded. Participants were divided into three groups based on their BMI values – lean, overweight and obese. The lean group comprised participants with BMI < 25, the overweight group comprised participants with 25 ≤ BMI < 30 and the obese group had BMI ≥ 30. Anthropometric (height and weight) and blood pressure measurements were taken at the time of collection, and a rapid HIV test done. We also had extensive other data about participants from previous engagements. The study was approved by the Human Research Ethics Committee (Medical) of the University of the Witwatersrand (M160121) and the Provincial Health Research Committee of the Province of Mpumalanga (MP2017TP22851).

To facilitate the participant recruitment and sample collection processes, comprehensive information sessions were held with the fieldworker on the study aims and its importance. This was crucial as the recruitment success could be reliant on the fieldworker’s ability to effectively communicate these to prospective participants. The fieldworker was also aided by training videos and experience gained from self-collecting personal stool samples to facilitate relatability to the collection process.

### Sample collection

Stool samples were collected from consented participants using DNA Genotek®‘s OMNIgene microbial collection and stabilization kit and sent to the laboratory. The stool samples were subsequently aliquoted into cryovials and frozen at − 80 degrees Celsius prior to DNA extraction.

### DNA extraction and sequencing

Frozen stool samples were thawed on ice. Genomic (total) DNA was extracted using Qiagen®‘s QIAmp Powerfecal DNA kit and sent to a dedicated core facility for the sequencing of the V3 –V4 hypervariable region of the 16S rRNA gene on the Illumina MiSeq® platform using 341F 5’-CCTACGGGNGGCWGCAG-3′ and 805R 5′-GACTACHVGGGTATCTAATCC-3′ as forward and reverse primers respectively [83].

### Sequence data analyses

The DADA2 (v1.10.1) pipeline [43] was used for pre-processing and performing quality control on the sequences. Briefly, the demultiplexed paired-end sequences were imported into DADA2. Based on the quality plots, the sequences were filtered with a maximum of expected errors of 2 and 4, and sequence lengths of 280 and 240 bases for the forward and reverse reads, respectively, with primers trimmed accordingly. The resulting reads were dereplicated and merged to obtain the full denoised sequence which was used in the creation of a count table containing the abundance values of sequence variants from the sampled data. Chimeras were subsequently removed, and the non-chimeric sequence table was utilized for downstream analyses.

### Taxonomic classification

The DADA2 implementation of the naïve Bayesian classifier methodwas applied in the assignment of taxonomies to the amplicon sequence variants using the *RDP trainset 16* DADA2-formatted reference set from the Ribosomal Database Project (RDP) [84] and a minimum bootstrapping parameter of 50, with pseudo-pooling.

### Alpha and Beta diversity analyses

The DADA2 output together with the sample metadata were imported into phyloseq [44] for diversity analysis. Based on the output from the pre-processing step, rarefaction was applied at a sampling read depth of 50,800 to allow for adequate capture of the observed microbial taxa richness in the cohort as diversity metrics are generally sensitive to sample read depths.

First, Shannon [47] and Chao1 [48] alpha diversity estimates for the samples were calculated. This measure was applied to a pairwise Wilcoxon rank sum (Mann-Whitney) test to assess whether the observed ASVs differed significantly (*p* < 0.05) between specified categories. Boxplots were generated to visualize the categorical differences based on the Shannon diversity values. Comparisons were done as indicated in Table 4.

**Table 4:** Group comparisons evaluated in this study

Groups	Targeted Evaluation	Sample Data (No. of Samples)
Bushbuckridge *vs* Soweto	Overall site differences	All samples - lean, overweight and obese (167)
Bushbuckridge *vs* Soweto	Compositional differences in the lean category between sites	Bushbuckridge and Soweto lean samples only (30)
Bushbuckridge *vs* Soweto	Compositional differences in the obese category between sites	Bushbuckridge and Soweto obese samples only (106)
Lean *vs* Obese	Cohort-wide BMI compositional categorical differences	Bushbuckridge and Soweto lean and obese samples only (136)
Lean *vs* Obese	Site-specific BMI compositional categorical differences	Bushbuckridge lean and obese samples only (87)
Lean *vs* Obese	Site-specific BMI compositional categorical differences	SWT lean and obese samples only (49)

Next, beta diversity between the samples was evaluated using Bray-Curtis dissimilarity distance matrices for PCoA [49] to generate relevant ordination plots. PERMANOVA analysis was done to test for differences between specified categories (Table 4).

### Differential abundance analyses

To evaluate differences in bacterial taxa abundance across BMI categories and sites, a negative binomial generalized linear model (DESeq2) [45] was used. Briefly, raw counts were modelled with a negative binomial distribution and internal adjustment done for “size factors”. This adjustment normalized for differences in sequencing depth between samples. Prior to analyses, the data was filtered to exclude taxa that was not observed more than three times in more than 5 % of the 167 samples. This cut-off was chosen with respect to the sample size and the general data sparsity to protect against ASVs with small mean and trivially large coefficients of variation across samples. This resulted in 1688 high abundance ASVs being included in this analysis. DESeq2 models were adjusted for potential batch effects, where applicable, and BMI for the overall site analysis. However, it is highly unlikely that substantial batch effects exist as 14 samples from the first batch that were re-sequenced and compared across the two sequence runs using Bray-Curtis measure indicate the absence of any potentially damaging batch effects (Supplementary Figure 2).

Statistical significance was determined by the Wald’s test with Benjamini-Hochberg corrected *p*-values and significant ASVs above a secondary alpha threshold of 0.1. The results are presented with Volcano plots (Fig. 5 and Supplementary Table 2).

### Marker taxa analyses

To establish the status of our sampled cohorts along the continuum of westernization, we sought to compare the relative abundances of proposed Western and non-Western marker taxa as compiled by a recent meta-analysis [67] with the corresponding values in our dataset. The proposed taxa can be used as markers of lifestyle and geographical origin in the chosen public datasets as well as in the South African cohorts.

A total of 23 studies [5, 58, 85–105] with benchmarked Western and non-Western datasets comprising 1763 samples were downloaded from the curatedMetagenomicData [61] repository. The downloaded count data was converted to an ExpressionSet object and imported into phyloseq [44] for downstream analysis. The data was sub-setted to include only the eight genera of interest – *Prevotella*, *Treponema*, *Bifidobacterium*, *Barnesiella*, *Akkermansia*, *Alistipes*, *Bacteroides* and *Parabacteroides*. The abundance counts were transformed to relative abundance values and filtered to retain only ASVs with mean abundance greater than zero. The data was subsequently split by westernization and 167 samples were randomly selected from each of the two groups and merged with the South African (RSA) dataset to give two groups (Western-RSA and non-Western-RSA) of 334 samples each. These two sample groups were utilized for both comparisons between the subsampled cMD and our combined cohort data.

For each group of data, 70% (234) of the samples were used as the training set for Random Forest analysis to compare the two datasets, with the remaining 30% (100) as the test data. Variable Importance Plots were used to visualize the results (Fig. 6). Abundance levels of the selected taxa were also tested for significant differences using the Kruskal-Wallis test (Table 5).

**Table 5:** Marker taxa analysis. Comparisons between the South African (RSA) cohorts data and benchmarked data sets from the curatedMetagenomicData (cMD). (a) cMD Western (W) data vs RSA data and (b) cMD non-Western (NW) data vs RSA data. The Kruskal-Wallis (KW) rank sum test was used in the calculation of the *p*-values

**A**				
**Genus**	**RSA Median**	**W-Median**	**W-log**_**2**_**FC**	***p*-values (KW)**
*Prevotella*	0.543	0.030	4.409	2.20E-16
*Treponema*	0.116	0.003	5.085	6.30E-01
*Bifidobacterium*	0.015	0.043	-4.139	3.09E-16
*Barnesiella*	0.049	0.033	-0.552	1.39E-01
*Akkermansia*	0.056	0.012	0.251	9.39E-01
*Alistipes*	0.040	0.118	-2.789	2.20E-16
*Bacteroides*	0.268	0.384	-1.183	1.08E-08
*Parabacteroides*	0.043	0.050	-0.719	7.21E-05
**B**				
**Genus**	**RSA Median**	**NW-Median**	**NW-log**_**2**_**FC**	***p*-values (KW)**
*Prevotella*	0.543	0.760	-4.685	1.02E-03
*Treponema*	0.116	0.001	1.638	5.18E-01
*Bifidobacterium*	0.015	0.097	-1.159	2.20E-16
*Barnesiella*	0.049	0.001	4.481	6.28E-14
*Akkermansia*	0.056	0.001	3.227	9.20E-03
*Alistipes*	0.040	0.008	3.917	3.59E-03
*Bacteroides*	0.268	0.020	4.243	2.20E-16
*Parabacteroides*	0.043	0.006	3.091	2.20E-16

### Feedback from participants

The follow-up survey was done on the first 100 participants at Bushbuckridge about 3 months after collection. The survey was conducted telephonically – each person was phoned at least three times. One person refused to participate, and 65 people agreed. The community engagement process is detailed in the Supplementary data section.

## Supplementary information

**Additional file 1: Supplementary Figure 1.** Beta diversity PCoA plots with Bray-Curtis dissimilarity measure. Combined Bushbuckridge and Soweto datasets indicating differences in (A) Cohort-wide and (B) Lean vs obese categories. Site-specific lean and obese sampled data in (C) Bushbuckridge and (D) Soweto. Ellipses represent a 0.95 confidence interval.

**Additional file 2: Supplementary Figure 2.** Batch-control test. To control for batch effects from different sequencing runs, 14 samples from the first batch were re-sequenced. Comparison of the samples from the two sequence runs using Bray-Curtis measure indicates the absence of any potentially damaging batch effects.

**Additional file 3: **Extended information on the community engagement process. **Supplementary Table 1**. Sample reads tracked through the pre-processing steps. **Supplementary Table 2**. Genera, associated *p*-values and log_2_ fold changes corresponding to phyla on the volcano plots in Fig. 5.

## Data Availability

The nucleotide sequence data analyzed in this study can be accessed at the ENA under BioProject PRJEB40733. The corresponding phenotype data has been submitted to the EGA (study EGAS00001002482) in terms of the data sharing policy of the Human Heredity and Health in Africa consortium (H3A) and is available by request to the independent H3A Data and Biospecimens Access Committee which will consider each case in terms of H3A policies and to protect participants data. The R code to reproduce statistical analyses is available at https://github.com/SBIMB/awimbpilot

## Abbreviations

BBR: Bushbuckridge
BMI: Body Mass Index
CAG: Community Advisory Group
cMD: CuratedMetagenomicData
DPHRU: Developmental Pathways for Health Research Unit
H3Africa: Human Heredity and Health in Africa
HDSS: Health and Demographic Surveillance Sites
HIV: Human Immunodeficiency Virus
KW: Kruskal-Wallis
MRC: Medical Research Council
PCoA: Principal Coordinate Analysis
RSA: Republic of South Africa
SWT: Soweto
T2D: Type 2 Diabetes
Wits: University of the Witwatersrand
