# Supplementary figures and images for: Gut microbiome profiling of a rural and urban South African cohort reveals biomarkers of a population in lifestyle transition
Source: BMC Microbiol. 2020 Oct 31;20:330. doi: 10.1186/s12866-020-02017-w (PMC7603784; doi:10.1186/s12866-020-02017-w)

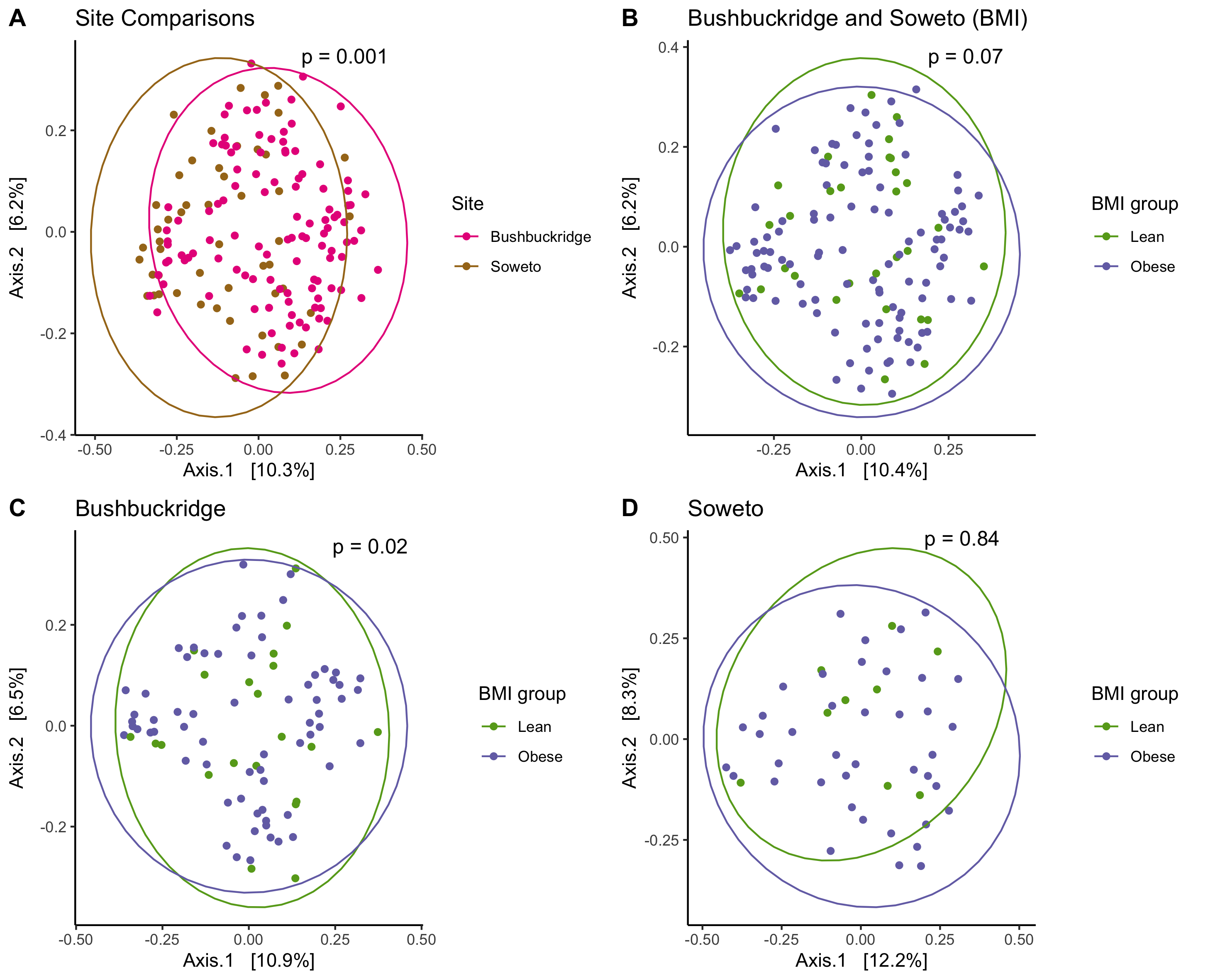

Supplement: Supplementary file 1 — Additional file 1: Supplementary Figure 1. Beta diversity PCoA plots with Bray-Curtis dissimilarity measure. Combined Bushbuckridge and Soweto datasets indicating differences in (A) Cohort-wide and (B) Lean vs obese categories. Site-specific lean and obese sampled data in (C) Bushbuckridge and (D) Soweto. Ellipses represent a 0.95 confidence interval. [file 12866_2020_2017_MOESM1_ESM.png]
