# Supplementary material for: Gut microbiome profiling of a rural and urban South African cohort reveals biomarkers of a population in lifestyle transition
Source: BMC Microbiol. 2020 Oct 31;20:330. doi: 10.1186/s12866-020-02017-w (PMC7603784; doi:10.1186/s12866-020-02017-w)
Supplement: Supplementary file 2 — Additional file 2: Supplementary Figure 2. Batch-control test. To control for batch effects from different sequencing runs, 14 samples from the first batch were re-sequenced. Comparison of the samples from the two sequence runs using Bray-Curtis measure indicates the absence of any potentially damaging batch effects. [file 12866_2020_2017_MOESM2_ESM.pdf]

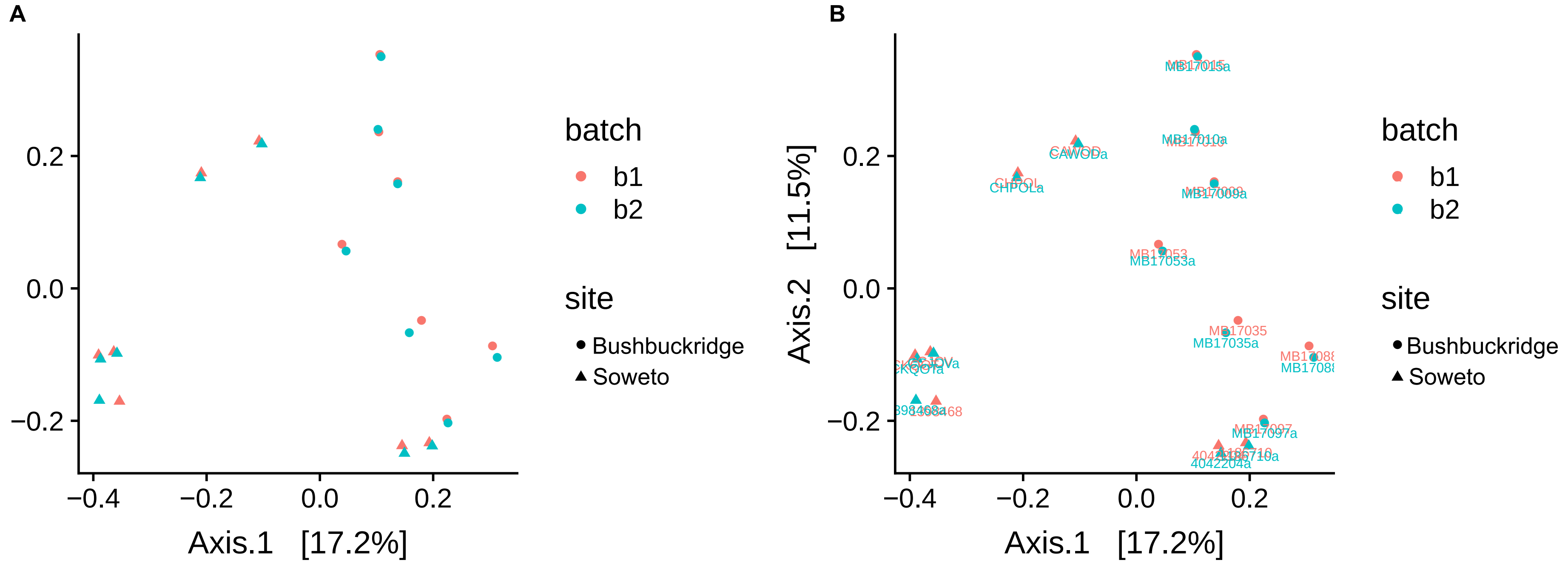
A
B
Axis.1 [17.2%]
Axis.2 [11.5%]
−0.4
−0.2
0.0
0.2
batch
b1
b2
site
Bushbuckridge
Soweto
MB17015a
MB17010a
MB17009a
CAWODa
CHPOLa
MB17053a
MB17035
MB17035a
MB17097a
4042204a
