# Supplementary material for: Gut microbiome profiling of a rural and urban South African cohort reveals biomarkers of a population in lifestyle transition
Source: BMC Microbiol. 2020 Oct 31;20:330. doi: 10.1186/s12866-020-02017-w (PMC7603784; doi:10.1186/s12866-020-02017-w)
Supplement: Supplementary file 3 — Additional file 3: Extended information on the community engagement process. Supplementary Table 1. Sample reads tracked through the pre-processing steps. Supplementary Table 2. Genera, associated p-values and log2 fold changes corresponding to phyla on the volcano plots in Fig. 5. [file 12866_2020_2017_MOESM3_ESM.pdf]

Supplementary Data for Oduaran, OH., Tamburini, FB., Sahibdeen, V., Brewster, R., Gómez-Olivé, FX., Kahn, K., Norris, SA., Tollman, SM., Twine, R., Wade, AN., Wagner, RG., Lombard, Z., Bhatt, AS., Hazelhurst, S. **Gut Microbiome Profiling of a Rural and Urban South African Cohort Reveals Biomarkers of a Population in Lifestyle Transition**. It contains:

1. Extended information on the community engagement process
2. Supplementary table 1 – Table of reads tracked throughout the pre-processing step.
3. Supplementary table 2 – Complementary table with corresponding genera to DESeq's volcano plots in Figure 5.

Community Engagement

The research team visited the Agincourt HDSS study area (rural Bushbuckridge site) during the planning phase of the study to discuss the proposed study with the Community Advisory Group (CAG), who are now permanent members of a study Research Advisory Group (RAG). Discussions with the RAG involved potential concerns of community members and reactions to collecting stool samples, as well as how to practically collect samples. It was deemed appropriate to attempt to collect stool in this population, provided that a detailed explanation of its intended usage would be provided by the trained field workers. Specifically, the RAG communicated that it was important to convey to participants that the stool would only be used solely for the proposed study, not be sold, and that any remaining sample would be disposed of after the of the study. In addition to interactive meetings with the RAG, graphical flyers with an overview of the research aims were made available to participants. An instructional video recording was provided to assist the training of field workers. It was also agreed upon that samples will be collected from participants in a culturally sensitive manner as available research stool collection kits are designed for use on Western toilets with plastic toilet seats, and most households in the study area use pit latrines with concrete or no toilet seats.

Sixty-five (65) responses were received from a follow-up survey done on the first 100 participants in the Bushbuckridge area to get their feedback on the process, with one refusal to participate. All 65 survey participants responded positively indicating an interest in future participation. Most found the instructions clear and useful, and they had not found participating embarrassing or unpleasant. Specifically, participants were asked three questions on the Likert scale (1=strongly agree, 5=strongly disagree). Questions and results were: Did you find the instructions clear (1.15),

did you find the instructions useful (1.46), did you find participation unpleasant or embarrassing (3.98). This survey was conducted telephonically, and we had a concern that the participants' responses may not have been entirely forthright. Several participants also asked about getting results back.

Following preliminary data analysis, the research team organized an interactive workshop with 18 CAG representatives on-site. The session reiterated the importance of the study, conveyed initial results, and solicited feedback from community members. Participants were encouraged to share their insights and learnings with a "concept map," a diagram that depicts relationships between different ideas, such as "bacteria", "health" and "disease. This provided the research team with a sense of the communal understanding of the study.

We struggled with deciding on the amount and type of information to report back, as DNA sequencing of stool is not a gold standard diagnostic test, and participants are used to receiving actionable health information as part of most studies at the HDSSs (e.g. HIV status or presence of a pathogen, like bilharzia). The HIV test, BMI and glucose level measurements done as part of the study were reported back. However, for microbiome data, science is not at the stage where meaningful results can be given, and so individual feedback would not be useful and may be potentially damaging and raise anxiety levels. This issue was raised with the RAG and understanding was shown. Nevertheless, we hope to work out a way of giving group feedback that be meaningful and if not useful at least interesting to our participants. In addition to the CAG's valuable advisory role to the success of the collection process, some of the participants present at

the feedback session noted the disposition of the fieldworker to be critical to their consenting to the study. We had a high participation rate in Bushbuckridge.

Supplementary Table 1

sampleID	input	filtered	dada_f	dada_r	merged	nonchim	% retained
FQOSV	221	28	23	8	2	2	0.9
BZEHG	261	30	20	24	17	17	6.5
ELHZE	59249	23257	22345	22531	20351	19560	33
DZPJR	78941	61259	60582	60904	58324	50812	64.4
JQKFV	79201	60111	59522	59645	57073	52180	65.9
CSK0E	87888	63962	62698	63277	59393	53158	60.5
HPRXQ	79678	60678	57951	59646	55608	54123	67.9
EZWQX	90765	69328	68230	68807	64861	55860	61.5
MB17034	86337	63630	62885	63165	61244	56491	65.4
CXCOE	95563	68646	67408	67955	64188	56559	59.2
EKZCV	84087	66570	65408	65927	62640	57030	67.8
HFZEJ	84861	66266	65289	65786	63096	57102	67.3
HVFJZ	87522	67541	65698	66633	62606	57240	65.4
CLLYS	89135	66075	63092	64725	60098	57683	64.7
3602219	97310	70537	70234	70249	68652	57728	59.3
BKHRQ	90134	70179	67339	68969	63296	58174	64.5
CRCOS	99531	71712	69497	70910	66337	58295	58.6
JLNGC	89603	68208	65491	67260	62912	58507	65.3
CTBOZ	90932	70148	68567	69524	65440	58917	64.8
CBSNC	88959	68463	65944	67349	62649	59133	66.5
MB17068	96090	71409	69689	70688	66596	59742	62.2
DZWHL	91654	68609	65757	67578	62777	60204	65.7
EDFHQ	89675	69433	67671	68654	64944	60250	67.2
MB17019	111382	81749	79828	80896	75914	60684	54.5
2123689	104000	77416	76489	76857	74556	62039	59.7
CKXOB	101589	74609	73086	73712	70370	62500	61.5
KDRZJ	91677	71626	69265	70478	66198	62715	68.4
CWIOI	102654	73311	71501	72378	68660	62744	61.1
3379474	101349	75209	74562	74752	72254	63087	62.2
JRWPH	96148	73850	71374	72997	68094	63299	65.8
MB17104	104096	77189	75820	76450	72351	63585	61.1
MB17074	102521	76201	74787	75508	72043	63851	62.3
4152135	102381	73013	69503	71691	66572	64048	62.6
HDWHX	103715	80127	77257	79013	74325	67194	64.8
MB17106	106678	75404	73635	74588	71678	67320	63.1
CWQ0Q	109737	77965	75003	76800	71818	67528	61.5
CQGFO	100291	76439	72884	75187	69887	67668	67.5
DFEZM	100008	78263	76831	77565	73610	67856	67.9
MB17100	106172	78724	77320	78068	74948	68055	64.1
CQMOC	112109	82733	81102	82017	77294	68125	60.8
KLHYD	105653	81166	76161	79622	72057	68876	65.2
CCPOB	104275	75241	73189	74410	71152	69863	67
MB17098	110346	80484	78144	79446	74919	69903	63.3
DKWHW	107960	81562	78946	80585	74967	69989	64.8
1186710	119766	87213	86097	86525	82376	70184	58.6

MB17006	114094	81610	80396	80910	77809	70412	61.7
CLNOT	116654	86921	85181	85898	81823	70981	60.8
CCLEW	105355	80733	77927	79559	75031	70988	67.4
DGXFV	109228	84485	81905	83483	78238	71174	65.2
1499095	121348	88510	87256	87818	83950	71784	59.2
BQNHZ	106631	84129	82051	83061	78091	71997	67.5
DGEOC	117073	87418	85600	86570	81266	73031	62.4
HGRSG	115600	85683	82190	84356	78362	74683	64.6
MB17015	118449	85896	82855	84544	79276	75782	64
MB17025	122457	88214	84994	86892	81741	76358	62.4
HEJHE	114424	87847	86247	86894	83058	76543	66.9
MB17101	128797	91562	88505	90372	84780	76795	59.6
2146745	131577	96220	93358	95157	87402	77024	58.5
MB17037	124490	90616	87148	89319	83290	77069	61.9
1333396	119574	88210	87292	87656	84892	77937	65.2
MB17105	124277	91850	89335	90726	85634	79313	63.8
MB17103	128835	94148	91337	92951	86381	79495	61.7
CAX0E	131406	92203	88998	90767	84640	79548	60.5
MB17010	130088	92238	89303	90824	84723	79820	61.4
FRGPC	118775	92046	88694	90951	85119	80374	67.7
MB17046	128297	92063	89635	90995	86412	81074	63.2
CVQON	136122	99881	97233	98567	91803	81264	59.7
1597674	132102	96229	93445	94931	89269	81871	62
DCGOV	136801	103049	100910	101898	96284	81873	59.8
MB17069	128834	93940	91378	92807	88062	82875	64.3
MB17102	137947	102623	100174	101529	95565	83352	60.4
CRXON	134707	95652	93151	94585	89463	83825	62.2
CKQOT	142884	103811	101098	102520	96175	85588	59.9
CDKOY	142448	104694	102933	103783	98922	86555	60.8
MB17026	140603	99778	97347	98771	93490	87292	62.1
3924929	146579	108039	105525	106896	100767	87705	59.8
CHPOL	142979	105367	103353	104284	98503	88253	61.7
MB17012	141069	103257	99731	101673	94412	88255	62.6
DDPOG	140812	105577	103842	104622	100031	88940	63.2
MB17018	142340	102461	99374	100964	95018	89053	62.6
CBZOI	146937	105007	101134	103519	96600	90176	61.4
CFI0B	140151	103323	100171	101862	96365	90491	64.6
MB17020	146433	106246	103966	105221	99170	91100	62.2
MB17022	144569	104954	101032	103387	97153	92392	63.9
MB17035	139597	109066	107268	108141	102530	92745	66.4
MB17009	144425	106449	102863	104877	98411	93387	64.7
DDWOM	146125	109124	107283	108053	103246	93490	64
2554637a	148496	107824	104137	106114	99079	93771	63.1
MB17004	145376	107878	105405	106813	101543	93788	64.5
DBT0G	146210	106496	103097	104942	98776	93843	64.2
MB17024	142795	106336	103887	105216	100091	94073	65.9

4971044	149527	110492	107192	108920	101473	95212	63.7
CAWOD	146133	108965	105745	107473	101423	95406	65.3
MB17011	155730	119301	117709	118315	112406	95471	61.3
MB17014	145991	109956	107366	108860	103103	95653	65.5
3956441	157781	117044	114570	115621	110128	96666	61.3
3480141	157012	118951	117237	118059	111938	96945	61.7
CCJOV	162002	117797	115615	116528	110823	97114	59.9
MB17016	153942	110179	106378	108391	102536	97136	63.1
2031518	158401	116063	113018	114789	107345	97380	61.5
MB17007	153383	111700	108881	110443	105064	97547	63.6
CFVOM	155322	111703	107802	109994	103279	97915	63
2266332	161177	116488	112096	114610	107088	98029	60.8
MB17033	148036	116348	115194	115653	110802	98378	66.5
1930043	155389	113739	110545	112224	105685	98421	63.3
DDZ0Q	158181	116061	113120	114877	108971	99146	62.7
MB17017	158388	115543	111503	113573	106809	102002	64.4
MB17003	159836	120407	117932	119298	113855	102262	64
DBGOS	161666	119608	116750	118222	111830	102475	63.4
DGV05	163368	120870	117245	119148	111447	102691	62.9
4233802	168833	123073	120330	121710	114722	102917	61
MB17013	164880	116367	113663	115010	109255	103812	63
4858889	167263	119620	116679	118139	111850	103863	62.1
DHHOH	163885	120798	116516	119094	111186	104629	63.8
MB17040	164417	129083	127289	128151	121900	105204	64
MB17038	167023	130113	128591	129150	122719	106359	63.7
MB17049	157620	125865	124457	125078	120475	106370	67.5
GWBHF	158344	119404	117115	118158	114174	109127	68.9
3663723	177608	126604	122088	124932	116400	109303	61.5
DEROK	176767	129145	126201	127920	121327	111776	63.2
MB17039	170703	131080	128255	129769	122307	111852	65.5
MB17085	171624	135866	133377	134567	127184	111865	65.2
MB17091	162502	128756	126150	127514	120417	112282	69.1
CBY0H	172399	128556	125033	127124	119885	112321	65.2
MB17062	163607	128163	125487	126614	119928	112596	68.8
MB17088	173673	136912	134212	135782	127832	113512	65.4
MB17036	174859	134932	132893	133584	127489	114079	65.2
MB17077	170345	135354	132636	134080	126740	114633	67.3
MB17071	174714	139039	137211	137894	131940	115289	66
MB17072	174424	136619	133667	135287	128698	116380	66.7
MB17070	169222	134915	133504	134044	128750	119632	70.7
MB17027	177523	137317	134212	135969	127980	120034	67.6
MB17030	177181	135903	132095	134286	126536	120353	67.9
MB17063	181513	143333	141097	142254	134917	121253	66.8
MB17094	180477	140738	137472	139058	131279	123311	68.3
MB17028	185251	143845	140281	142135	134055	124518	67.2
MB17066	190850	150096	147992	148765	140834	124699	65.3

MB17050	194435	152281	147071	150181	138099	127985	65.8
MB17083	189949	146771	142289	144635	135218	128085	67.4
MB17051	198862	154044	150697	152382	144125	128548	64.6
MB17056	191569	151059	147750	149616	140951	129497	67.6
MB17055	196883	154371	152366	153310	145918	129518	65.8
MB17076	187526	145907	142889	144494	137676	129583	69.1
MB17031	188405	146317	140784	144109	134874	129823	68.9
MB17080	199543	150525	145080	148297	138137	130264	65.3
MB17057	188453	148631	144934	146885	138785	130916	69.5
MB17093	195807	155302	151922	153801	144480	130996	66.9
MB17073	187906	149353	146479	147996	140918	131684	70.1
MB17081	192056	152849	150203	151388	144535	131885	68.7
MB17092	193078	153367	149953	151866	143145	131937	68.3
MB17043	193051	154712	152042	153234	145241	133495	69.2
MB17078	194868	151560	146099	149332	139501	133539	68.5
MB17082	202218	157475	152370	155408	145108	136915	67.7
MB17096	207652	160900	157158	158967	148987	137091	66
MB17061	205434	158393	154284	156690	147234	137305	66.8
MB17045	191882	154615	151597	153264	145756	137571	71.7
MB17075	206904	160792	156827	159085	149002	138152	66.8
MB17097	198707	156669	152886	154924	146481	139126	70
MB17054	216080	168465	165331	166967	157572	139749	64.7
MB17079	199205	154322	150052	152453	144979	139869	70.2
MB17053	204928	161070	156559	159132	148575	140004	68.3
MB17041	205535	159110	154241	157087	147719	140273	68.2
MB17064	208836	163161	158968	161186	151477	140677	67.4
MB17044	206910	162028	157930	160081	150472	140686	68
MB17067	215352	166591	163325	164818	154835	141225	65.6
MB17059	213035	165993	162611	164283	154094	141649	66.5
MB17058	221285	177493	174497	176162	167135	146469	66.2
MB17047	218450	166782	161348	164443	153834	146704	67.2
MB17042	214336	166872	161120	164305	153684	147900	69
MB17052	224349	179346	175726	177605	168800	154124	68.7

Supplementary Table 2A

Phylum	Genus	log2FoldChange	padj
Firmicutes	Faecalibacterium	-2.948289946	0.030143364
Firmicutes	NA	-3.164057658	0.030143364
Firmicutes	Faecalibacterium	-4.283834853	0.01591297
Proteobacteria	Vampirovibrio	-27.75261479	8.49E-14
Firmicutes	Ruminococcus	-10.7767581	5.18E-05
Tenericutes	Anaeroplasma	-5.670916745	0.006513525
Firmicutes	Streptococcus	4.089000849	0.052075231
Proteobacteria	Sutterella	-25.21401389	1.43E-11
Firmicutes	NA	-8.829621222	0.002563705
Bacteroidetes	Prevotella	-6.063410789	0.025198733
Proteobacteria	Vampirovibrio	-24.72778668	1.53E-12
Bacteroidetes	Alistipes	-30	8.49E-14
Firmicutes	Acetanaerobacteriu	10.6204115	0.03345809
Firmicutes	Gemmiger	-5.74640603	0.065463474
Firmicutes	Flavonifractor	-3.851839866	0.01591297
Proteobacteria	Parasutterella	-11.70775137	0.065056778

Supplementary Table 2B.

Phylum	Genus	log2FoldChange	padj
Bacteroidetes	NA	23.07789071	0.004363688
Firmicutes	Clostridium_IV	23.80568491	2.59E-05
Proteobacteria	Sutterella	23.13578032	1.22E-06
Firmicutes	NA	-9.076755715	0.037057223
Firmicutes	Ruminococcus	20.80380598	0.000771925
Firmicutes	Catenibacterium	21.51908773	0.000428028
Bacteroidetes	Barnesiella	16.61820089	0.055699717
Bacteroidetes	Prevotella	17.58681342	0.037057223
Bacteroidetes	Prevotella	19.04868921	0.024275008
Firmicutes	NA	21.69196387	0.004363688
Bacteroidetes	Prevotella	17.57697901	0.055699717
Proteobacteria	NA	-27.56742608	0.004363688
Bacteroidetes	Prevotella	20.8689034	0.018956964
Proteobacteria	NA	19.59851144	0.004363688
Firmicutes	Catenibacterium	23.08314541	0.004242592
Bacteroidetes	Bacteroides	21.00415968	0.043570491
Proteobacteria	NA	20.97086388	0.050245214
Bacteroidetes	Prevotella	18.75869803	0.024397052
Firmicutes	NA	20.39809781	0.005193846
Firmicutes	Phascolarctobacteri	19.34589557	0.014581396
Firmicutes	Phascolarctobacteri	20.07233943	0.004363688
Bacteroidetes	Bacteroides	-27.98005364	0.004363688
Firmicutes	Ruminococcus	19.39642463	0.024397052
Firmicutes	NA	18.88135078	0.0130629
Firmicutes	Roseburia	19.59342593	0.0130629
Bacteroidetes	Bacteroides	19.85557246	0.050245214
Firmicutes	NA	-26.39285726	0.0130629
Firmicutes	Dialister	-26.05691265	0.0144722
Proteobacteria	Oxalobacter	19.6668361	0.005544641
Firmicutes	NA	18.8726824	0.004363688
Actinobacteria	Bifidobacterium	21.43003821	0.043570491
Bacteroidetes	Alistipes	20.19017854	0.082956833
Proteobacteria	NA	-24.90358657	0.00379038
Bacteroidetes	Parabacteroides	19.70540334	0.092649195

Supplementary Table 2C

Phylum	Genus	log2FoldChange	padj
Firmicutes	Ruminococcus	-26.22798711	1.28E-08
Firmicutes	NA	-24.70095282	1.80E-06

Supplementary Table 2D

Phylum	Genus	log2FoldChange	padj
Firmicutes	NA	21.85066972	1.63E-06
Bacteroidetes	Prevotella	8.498127802	0.04874207
Firmicutes	NA	7.035533845	0.035013111
Firmicutes	Intestinimonas	5.317561726	0.013007126

Supplementary Table 2E

Phylum	Genus	log2FoldChange	padj
Bacteroidetes	Prevotella	7.955170427	0.013817339
Bacteroidetes	Prevotella	7.806335946	0.061021924
Firmicutes	Clostridium_IV	11.67724401	0.026351365
Verrucomicrobia	Akkermansia	23.14067417	0.002987482
Bacteroidetes	Prevotella	22.98289739	0.000871992
Proteobacteria	Sutterella	22.81338015	1.23E-05
Proteobacteria	Vampirovibrio	23.60461056	0.000667746
Firmicutes	Ruminococcus	10.48231218	0.051180108
Bacteroidetes	NA	21.74180625	0.042649685
Firmicutes	Phascolarctobacterium	9.921809639	0.046115747
Firmicutes	Catenibacterium	22.0300666	1.81E-05
Bacteroidetes	Prevotella	22.27959402	0.000242195
Bacteroidetes	Prevotella	21.25744432	0.019972473
Firmicutes	NA	22.10963911	0.000723139
Bacteroidetes	Prevotella	22.3751762	0.000237212
Bacteroidetes	Prevotella	21.23611047	0.001137077
Firmicutes	NA	19.96218611	0.004182473
Bacteroidetes	Prevotella	21.4358074	0.005805978
Bacteroidetes	NA	21.65551294	0.015792607
Firmicutes	NA	21.73589394	0.006424543
Firmicutes	NA	20.96011721	0.006424543
Proteobacteria	NA	21.58143898	0.000606761
Proteobacteria	Sutterella	21.32126346	0.000667746
Bacteroidetes	Prevotella	22.06118089	0.000968635
Firmicutes	NA	20.33883533	0.000764727
Proteobacteria	Sutterella	20.97976298	0.001490284
Bacteroidetes	Prevotella	21.26448207	0.000488866
Bacteroidetes	Parabacteroides	20.71567168	0.005805978
Bacteroidetes	Prevotella	21.21881664	0.018376084
Firmicutes	NA	20.13576996	0.076543027
Bacteroidetes	Prevotella	20.84337422	0.01362984
Firmicutes	Phascolarctobacterium	20.14788351	0.002240367
Firmicutes	NA	18.8634611	0.013842708
Bacteroidetes	Alistipes	18.52687909	0.090741918
Firmicutes	Acetanaerobacterium	18.85665737	0.040420128
Firmicutes	NA	17.85217018	0.042649685

Supplementary Table 2F

Phylum	Genus	log2FoldChange	padj
Proteobacteria	Escherichia/Shigella	3.456849448	2.51E-05
Bacteroidetes	Prevotella	3.274300852	0.027676485
Firmicutes	Oscillibacter	3.558253002	0.075784277
Bacteroidetes	Prevotella	3.622352002	0.029384796
Firmicutes	Ruminococcus	3.522695798	0.09282651
Firmicutes	NA	-3.197232699	0.060222044
Bacteroidetes	Prevotella	5.225332346	0.030918399
Firmicutes	NA	3.141557796	0.039689525
Firmicutes	Clostridium_XlVa	5.709221797	0.034589787
Bacteroidetes	Prevotella	23.59680598	3.51E-14
Firmicutes	Fusicatenibacter	-1.372436177	0.09282651
Firmicutes	Clostridium_XlVa	1.967631098	0.029542144
Firmicutes	Oscillibacter	3.554312373	0.057210422
Bacteroidetes	Prevotella	6.371480701	0.08584636
Firmicutes	Sporobacter	3.714138311	0.030918399
Firmicutes	NA	-1.400252229	0.080599776
Bacteroidetes	Prevotella	9.176648127	0.000213391
Lentisphaerae	Victivallis	-3.273273869	0.078058153
Firmicutes	Clostridium_IV	-2.082113778	0.010623531
Proteobacteria	Haemophilus	3.297603024	0.022765633
Firmicutes	NA	2.551949643	0.078058153
Proteobacteria	Haemophilus	4.587783366	0.027676485
Bacteroidetes	Parabacteroides	21.87189622	3.51E-14
Firmicutes	Intestinimonas	4.471077903	0.006704835
Firmicutes	Clostridium_XlVb	4.584736133	0.028085092
Proteobacteria	Oxalobacter	4.462329106	0.075784277
Firmicutes	Clostridium_XlVa	20.10712772	3.51E-14
Firmicutes	Streptococcus	2.556688889	0.09282651
Bacteroidetes	Parabacteroides	-25.05231647	3.51E-14
Firmicutes	NA	-3.704455674	0.027676485
Proteobacteria	Haemophilus	4.383920391	0.08584636
